# Investigation of 200 anthropogenic activities in a representative alpine peatland in the Altay Mountains, northwestern China

**DOI:** 10.1007/s11356-024-33498-1

**Published:** 2024-05-06

**Authors:** Nana Luo, Rui Yu, Bolong Wen, Xiaoyu Li, Qilin Zhang, Xiujun Li

**Affiliations:** 1 Key Laboratory of Wetland Ecology and Environment, Northeast Institute of Geography and Agroecology, Chinese Academy of Sciences, Changchun, 130102 China; 2 University of Chinese Academy of Sciences, Beijing, 100049 China

**Keywords:** Alpine peatlands, Anthropogenic impacts, Polycyclic aromatic hydrocarbons, Black carbon, Altay Mountains

## Abstract

Peatlands records can be used to reconstruct and understand the history of environmental evolution, as well as a more accurate reflection of human activities. The black carbon (BC) and polycyclic aromatic hydrocarbons (PAHs) are ideal natural archives of anthropogenic activities. To identify the information of anthropogenic activities recorded by peatlands in the middle and high latitudes of the alpine mountains in the arid and semi-arid regions of China. this study analyzed the concentrations of BC, δ^13^C ratios of BC, PAHs, and molecular diagnostic ratios of PHAs (including Benzo(a) anthracene (BaA), Chrysene (Chr), fluoranthene (Flt), anthracene (Ant), phenanthrene (Phe), Benzo(a) pyrene (BaP), and pyrene (Pyr) in a 30-cm peat profile from the Altay Mountain, northwestern China. Our results revealed concentrations of BC from 11.71 to 67.5 mg·g^−1^, and PAHs from 168.09 to 263.53 ng·g^−1^. The δ^13^C_BC_ value ranged from − 31.37 to − 26.27‰, with an average of − 29.54‰, indicating that the BC mainly comes from biomass combustion. The ratios of BaA/(BaA + Chr), Flt/(Flt + Pyr), and Ant/(Ant + Phe) exceeded 0.35, 0.5, and 0.1, respectively, revealing that the PAHs pollutants mainly originated from the combustion of biomass and fossil fuel burning. Furthermore, based on these findings and our knowledge of social development in Altay, industrial transport and tourism have influenced the emission, transport, and deposition of BC and PAH in peatlands in the Altay mountains since the 1980s. After 1980, pollutant concentrations decreased with the implementation of environmental policies. The results not only reveal the influence of anthropogenic activities on the sedimentary characteristics of peatlands in the Altay Mountains, but also provide an important theoretical basis for the conservation of fragile mountain peatlands.

**Supplementary Information:**

The online version contains supplementary material available at 10.1007/s11356-024-33498-1.

## Introduction

Peatlands are a good geological-historical archive for recording climate change, which can be used to reconstruct the history of regional climate change and provide a scientific basis for predicting future climate and environmental changes (Blackford 2000; Biester et al. 2004; Drollinger et al. 2019; Yu et al. 2023). Northern peatlands hold about a third of the global reduced carbon (C) pool (Glooschenko et al. 1986) and hence have essential roles in the global carbon cycle and the climate system (Pratte et al. 2018). In the last 100 years, environmental pollution caused by anthropogenic activities has become more noticeable and has led to dramatic changes in regional climate, especially causing severe damage to sensitive and fragile peatland ecosystems in the middle and high latitudes of northern China (Cong et al. 2016; Luo et al. 2021).

High temperatures, influenced by climate change, tend to cause an increase in the frequency of wildfires, which destroy the surface vegetation of peatlands and subsequently produce a large amount of incomplete combustion products, namely black carbon (BC), which is chemically stable and can be preserved in the soil for a long time (Gao et al. 2018; Leifeld 2018; Leng et al. 2018). In order to better understand the BC emission patterns and their impact on climate change, work has been gradually carried out around the world to reconstruct BC deposition fluxes in historical periods from BC contents in different sediment archives (Ramanathan and Carmichael 2008; Zhu et al. 2017). Recently, fossil fuel combustion, garbage, and straw burning from industry, transportation, and agriculture have significantly altered the global carbon cycle through the emission of greenhouse gases and BC particles (Kuhlbusch 1995; He et al. 2016; Hu et al. 2020). The sources of BC are mainly natural and anthropogenic, where natural sources mainly include volcanic eruptions, forests, and fires; anthropogenic sources include coal and anthropogenic sources include coal and biomass combustion, straw, domestic waste burning, industrial coal combustion, coke production, iron and steel smelting, and motor exhaust (Preston and Schmidt 2006; Cai 2011). PAHs and other organic pollutants are often co-emitted with BC from natural or anthropogenic sources (IARC 1983; Menzie et al. 1992; Bai et al. 2008; Ray et al. 2012). Typically, a PAH concentration of 0 to 10 ng·g^−1^ in the soil reflects natural sources of pollution (e.g., forest fires) (Edward 1983; Giesy et al. 2010; Bao et al. 2020). Due to their difficult degradation and hydrophobicity, PAHs can accumulate in soil and sediments for a long time (Chen et al. 2017; Chang et al. 2018). Generally, a high concentration of PAH with low aromaticity originates from combustion (Yunker et al. 2002; Yang et al. 2021). This is a moderate pollution. More than 1000 (ng·g^−1^) is moderately polluted (Hu et al. 2017).

Due to the special topography of Altay Mountains in northern Xinjiang, the water resources are abundant and the climate is cold, while these natural conditions provide a favorable guarantee for the formation and development of peatlands in high-altitude permafrost areas, and are mainly distributed in ecologically sensitive areas such as river banks and inland lakeshore (Zhang et al. 2018). In recent years, due to an increase in human disturbance, the peatland patches in the Altay Mountains show fragmentation, the area of the peatlands has decreased, and the peatlands development has been degraded (Luo et al. 2020). The impacts of human activities on Altay peatlands are poorly documented. Accordingly, environmental proxies are necessary to evaluate a century of the impact of human activities on the peatlands. BC and PAHs are produced by incomplete combustion of biomass and fuel, which widely exist and can be deposited in the environment for hundreds of years (Gabov et al. 2020). For these reasons, BC and PAHs are indicators that can be used to resolve past degrees of human activity (Zhang et al. 2017; Bao et al. 2023). Previous studies have used peatlands deposition and storage to analyze the status of the Altay peatlands (Zhang et al. 2020), the majority of which have focused on the effects of Holocene climate change on the development of peatlands (Ming et al. 2010; Wang 2017; Su et al. 2017; Rao et al. 2019).

Dating the start of intensive anthropogenic influence on ecosystems is important for identifying the conditions necessary for ecosystem recovery. However, few studies have focused on determining when anthropogenic influences on wetland began through sedimentary archives. To fill this critical gap in our knowledge, this study evaluates anthropogenic activities through BC and PAHs and establishes a correlation between peatland properties and the concentration of BC and PAHs. The results can reveal the effects of human activities on the peatland ecosystem and enrich the research content of the peatland deposition process.

## Materials and methods

### Site description

The Altay Mountains in China are located in the northernmost part of the Xinjiang Uygur Autonomous Region, between 85°31′37″ to 91°1′15″E and 46°30′35″ to 49°10′45″N. The climate is a cold continental temperate zone, and mean temperatures vary between − 12 and − 16 °C in January, and do not exceed 16 °C in July. Precipitation occurs irregularly throughout the year—primarily from June to August—with the mean annual ranging from 600 and 350 mm, from west to east, respectively. Special topographic features, abundant water resources, and the cold climate of the Altay Mountains of Xinjiang make peatland resources abundant. These conditions are favorable for the formation and development of peatland mounds in high-altitude permafrost regions (Zhang et al. 2018; Luo et al. 2022). The Heihu peatlands fall under the jurisdiction of the Kanas Nature Reserve. The peatland mounds in this area are low and elongated, generally 2 to 3 m tall, and connected to each other up to 20 m in length. A large amount of surface runoff (via streams and ditches) in the small basins south and southwest of Heihu immerses most of the peatland mounds in water; this causes slow peatland mound development and a collapse at the edge of these mounds. Therefore, the peatland mounds in this area are low with regular collapses and degradation.

**Fig. 1:**
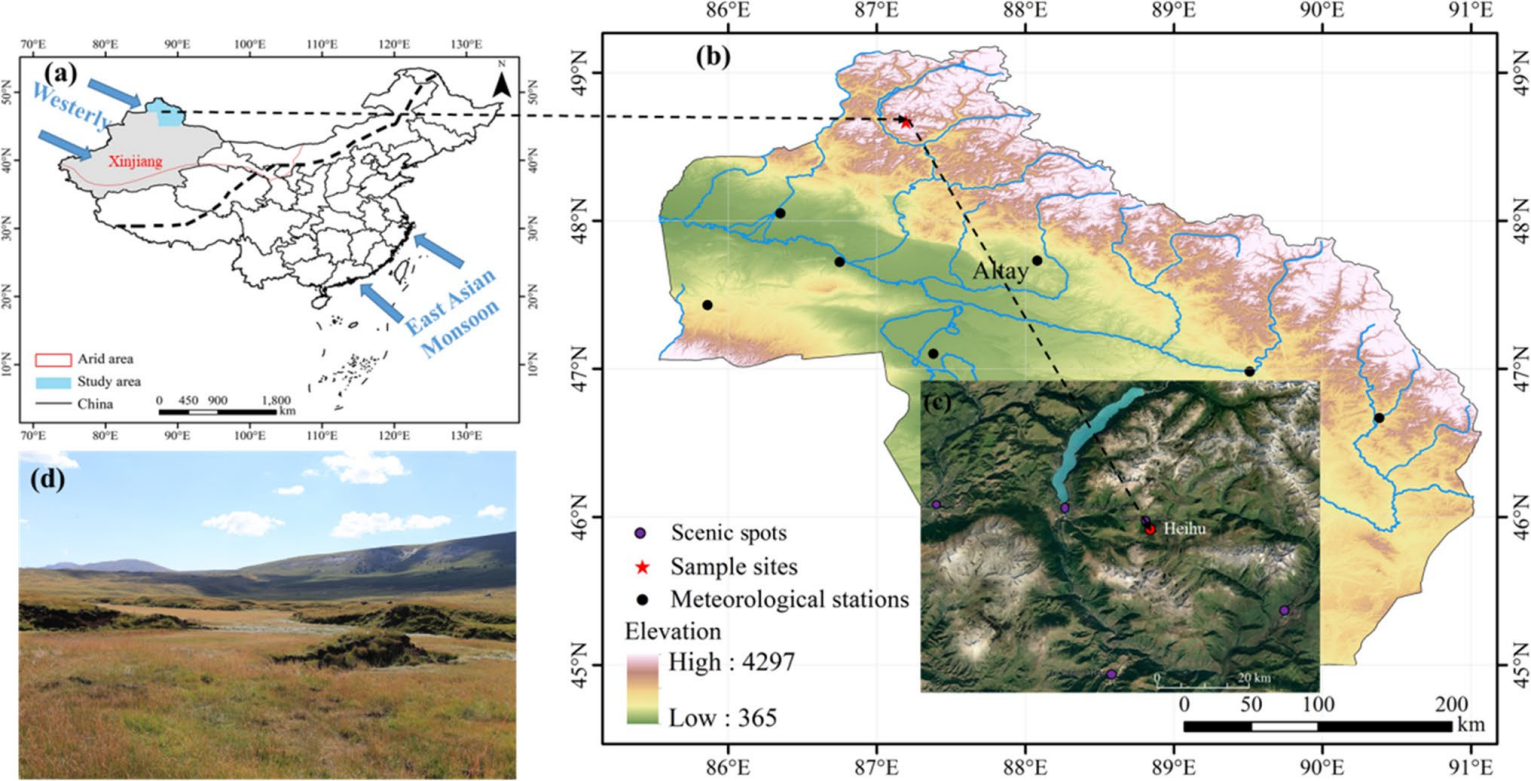
**a** Map of Xinjiang province in northwest China (created using ArcGIS version 10.4). **b** Geographical map showing the sampling site in the Altay Mountains in northwest China (also created using ArcGIS version 10.4 to draw sampling points in the study area). **c** Satellite image of the Heihu peatland. **d** Photograph of the peatlands during the summer they were (photo by Nana Luo)

Peatland core samples were collected in August 2019 at sampling sites located at Heihu (48°41′05.50″ N, 87°11′06.79″ E) at an altitude of 2190 m. Figure 1 provides details about the sampling sites. To prevent disturbance, 30-cm blocks were dug, and then 1-cm section was prepared. All samples were kept in polyethylene bags.

### Physicochemical analysis

#### ^210^Pb and ^137^Cs dating

Samples in the amount of 5 g were analyzed to measure ^210^Pb, ^226^Ra, and ^137^Cs using a low-background γ-ray spectrometer with a pure Ge semiconductor (ORTEC USA). The radioactivity of ^210^Pb was captured according to gamma emissions at 46.5 keV. Moreover, ^226^Ra was captured based on 295 keV and 352 keV γ-ray propagation of nuclide ^214^Pb after keeping the samples for 3 weeks in sealed containers. Meanwhile, Cs-137 was analyzed based on the 662 keV photopeak. The calculated half-life of ^137^Cs was 50,000 s and for ^210^Pb was 86,000 s, indicating that the measurement precision was ± 5% and ± 10%, respectively. During analyses, it was assumed that supported ^210^Pb was in equilibrium with ^226^Ra, and unsupported ^210^Pb activities were obtained based on the difference between the total ^210^Pb and the supported ^210^Pb activity (Bao et al. 2010).

#### Physicochemical properties and BC analysis

In the present study, different parameters including water content, bulk density, and the ash content of the peatland cores were analyzed. The method developed by Gao et al., (2014) was used to obtain the BC content in peatlands soils. In this regard, it is necessary to remove inorganic carbon and treat samples for 20 h in 10 mL of 1 M hydrochloric acid (HCl). After digestion, the contents were centrifuged and the residue was digested for 20 h with 10 mL acid mixture (3 mol/L HCl + 22 mol/L HF, a volumetric ratio of 1:2). Afterwards, the residue was soaked in 1 M HCl (10 mL) for 10 h. Accordingly, the residue contained organic matter, kerogen, and BC. A 0.1 M solution of sodium hydroxide (NaOH) was added to this residue to eliminate humic acid, then the residue was soaked in a mixture of 0.1 M K_2_Cr_2_O_7_ and 2 M sulfuric acid (H_2_SO_4_) for 60 h to remove non-pyrogenic organic carbon (NPOC). A hot water bath was used to maintain all solution temperatures at 55 °C. After the final sample residue underwent combustion at 960 °C, residual carbon was analyzed using a continuous-flow isotope ratio mass spectrometer (CF-IRMS), consisting of a Flash series 200 elemental analyzer (EA) and a mass spectrometer.

#### δ^13^C_BC_ analysis and PAHs analysis

After sample pretreatment, the residual carbon was quantified, with charred wood used as the BC reference material (Hammes et al. 2006). samples were used to calibrate the instruments and monitor the working conditions. Carbon isotope ratios (δ^13^C) were expressed relative to the standard Pee Dee Belemnite. Samples were naturally air-dried and ground through a 70-mesh sieve. The method of Cong et al., (2022) was followed: 5 g of dried peat samples were weighed and added to 20 g of anhydrous sodium sulfate (Na_2_SO_4_) in 200 mL of a 1:1 (v/v) hexane/acetone solution which was digested at 35 °C with the Soxhlet extraction method. The solution was then reduced to 5 mL, and copper powder was used to remove sulfur. The remaining solution was run on a Florisil Na_2_SO_4_-silica gel- Na_2_SO_4_ column using 40 mL of pentane solvent (to remove aliphatic ethers) leaching and solvent-exchanged with hexane in a rotary evaporator to reach a final volume of 1 mL.

### Quality assurance/quality control

In order to avoid potential contamination during the experiment, all containers were rinsed with ultrapure water. The Agilent 7890A-GC/5975C-MSD was used for GC–MS analysis. The chromatographic column was HP-5 fused silica capillary column (30 m × 0.25 mm × 0.25 µm), the temperature of the sample inlet is 300 °C, the initial temperature of the column is 50 °C, hold for 5 min, raise the temperature to 220 °C at the rate of 4 °C/min, and raise the temperature to 320 °C at the rate of 2 °C/min, hold for 25 min; The carrier gas is helium, the flow rate is 1 cm^3^/min, and the scanning mode is full scanning and selective ion scanning. PAHs compounds were identified according to retention time of chromatogram and GC–MS mass spectrometry database; Calculate the PAHs content according to (d10-Phenanthrene) standard sample.

### Statistical analysis

Descriptive statistical analyses were conducted to calculate the means, ranges, and standard deviations of the peatlands parameters. All data sets were checked for normality of distribution. Moreover, linear regression analysis was used to investigate the relationship between the BC content and TOC, PAHs in Heihu peatlands; Pearson correlation coefficients were calculated to evaluate the relationships among the individual parameters, respectively. These procedures were performed using SPSS 22 software package. Statistical significance was determined at the *P* = 0.05 level except if indicated differently.

## Results

### Chronology

**Fig. 2:**
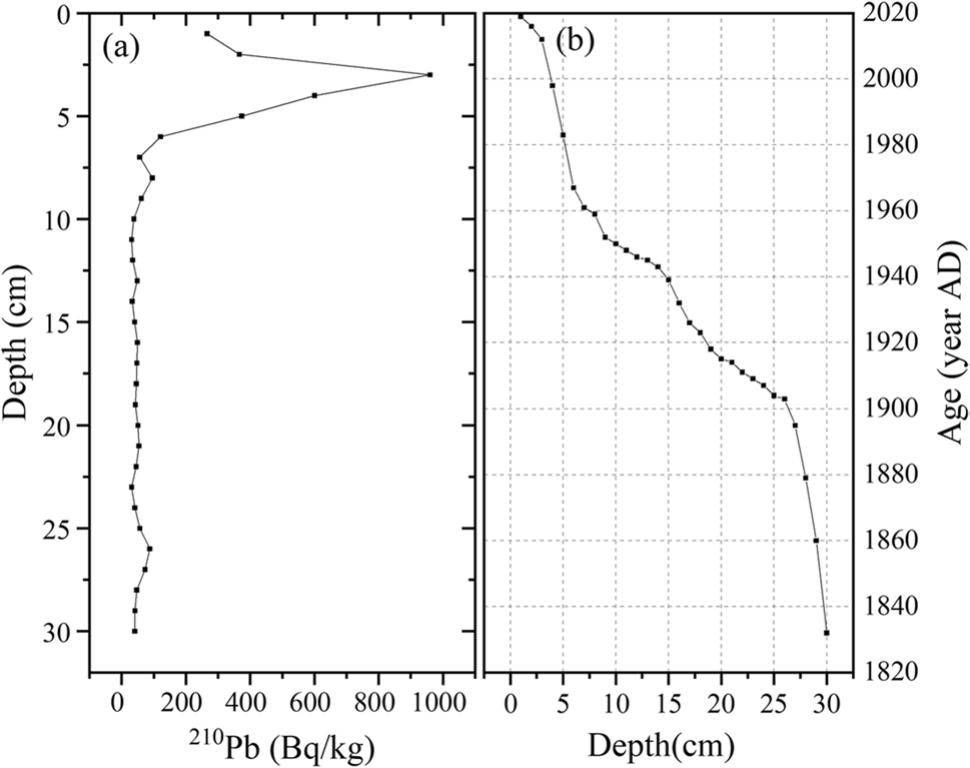
Sediment dating results obtained from the CRS model. **a** Radioactivity results for ^210^Pb. **b** The CRS calculated age of the Heihu peatland

Figure 2 plots ^210^Pb signatures over a sedimentary record that covers a period of 186 years, from 1832 to 2018. Based on the performed analyses and deposition rates, the peatland profile was divided into three periods: 1832–1910 (23–30 cm), 1910–1980 (22–6 cm), and 1980–2018 (6–1 cm). The average sediment deposition rate in these periods was 0.03, 0.02, and 0.3 cm year^−1^, respectively. The mean sedimentary and peatland accumulation rates were 0.028 cm year^−1^ and 0.004 g·cm^−2^ year^−1^ in the Heihu peatlands.

### Variation of BC content and δ^13^ CBC

**Fig. 3:**
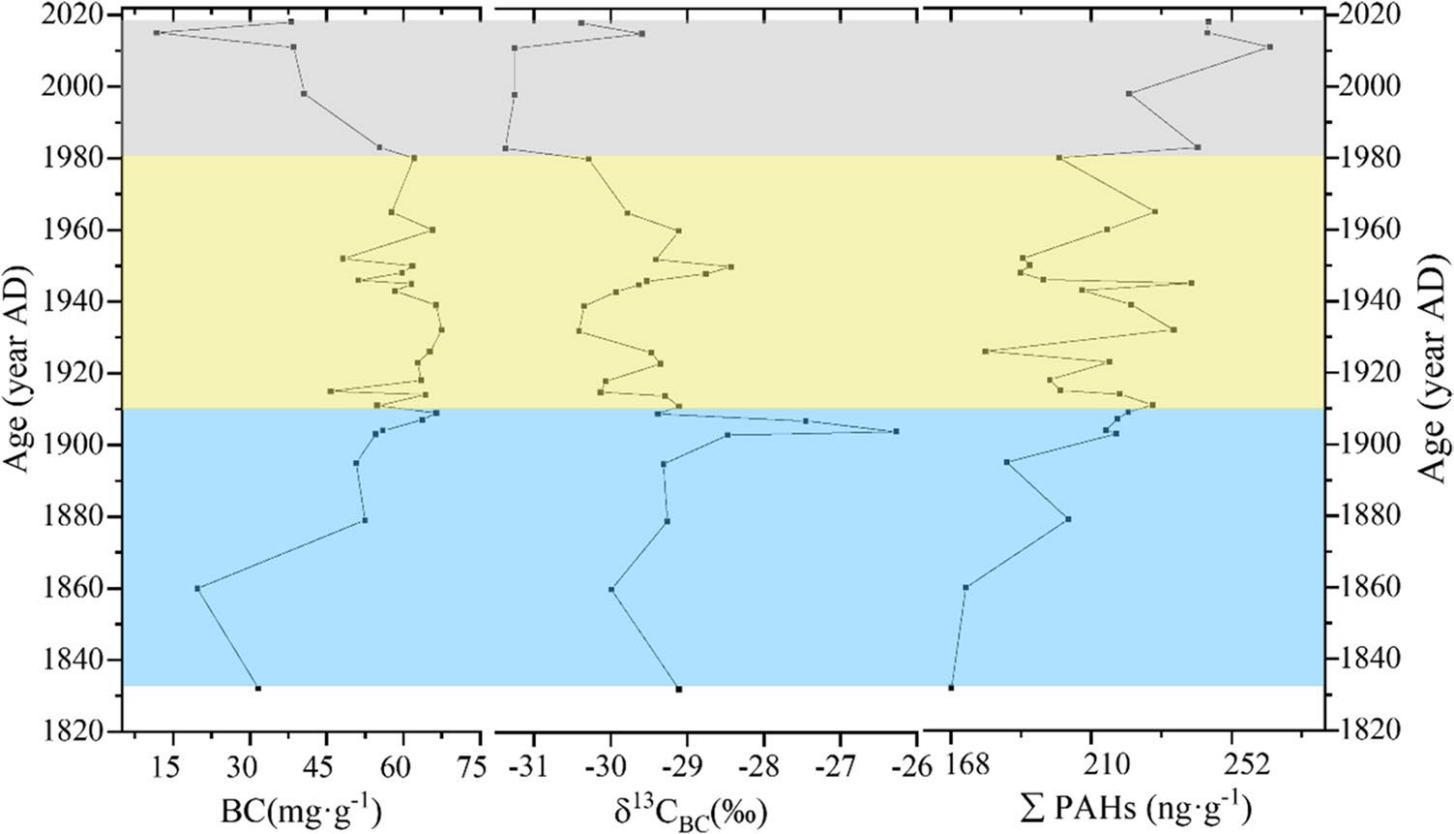
The contents of BC, δ^13^C_BC_ ratios, and the total PAH content in Heihu peatland

The average BC content in the Heihu peatland core was 53.19 mg g^−1^, with a range of 11.71 to 67.5 mg g^−1^. The highest level of BC (67.5 mg g^−1^) was measured at 16 cm, and the lowest (11.71 mg g^−1^) at 2 cm (Fig. 3). Combined with the chronological results, we divided core δ^13^ C_BC_ data into three ranges: data prior to 1910, data from 1910 to 1980, and data from after 1980. In general, BC increased from 19.71 to 66.48 mg g^−1^ before 1910, decreased from 1910 to 2012 (average 60.05 mg g^−1^), and increased after 2012. The value of δ^13^C_BC_ ranged from − 31.37 to − 26.27 ‰ with a mean of − 29.54 ‰ (Fig. 3). The highest value was − 26.27 ‰ at 25 cm, and the lowest value was − 31.37 ‰ at 5 cm. From 1 to 10 cm (associated with the years c. 1960 to 2018), δ^13^C_BC_ increased; however, δ^13^C_BC_ decreased between 11 and 16 cm (c. 1920 to 1960) and then increased to a maximum (− 26.27‰; c. 1904) before decreasing. Combined with the age-depth model, δ^13^C_BC_ values increased during the period of 1832 to 1904, then decreased from 1910 to 1980. δ^13^C_BC_ values showed a clear and consistent increase after 1980. Together, these data indicate that the proportion of biomass used in combustion had decreased prior to 1980, with a subsequent increase from industry sources.

### PAH concentrations and composition

The average PAH concentrations (ng·g^−1^) and the total PAH standard deviation from different periods are shown in Fig. 3 and Table S1. We found that PAHs clearly decreased as peatland depth increased. The total PAH concentration ranged from 168.09 to 263.53 ng·g^−1^, with a mean value of 212.25 ng·g^−1^. The highest concentrations appeared in the 3 cm layer (263.53 ng·g^−1^) and the 13 cm layer (239.97 ng·g^−1^). The lowest value appeared in the 30 cm layer (168.09 ng·g^−1^). Peatland PAHs increased before 1910, but from 1910 to 1980, they decreased slightly with a low value of 178.35 ng·g^−1^ and a high value of 239.97 ng·g^−1^. After 1980, PAH concentrations generally increased. Moreover, PAH concentrations from 1980 to 2018 (*n* = 5; 6 to 1 cm depth) were higher than those from 1910 to 1980 (*n* = 21; 22 to 6 cm depth) (Fig. 3). The PAH concentration in sediment is used to assess the pollution level and classify it into one of four categories (Table S2). According to Table S2, the study sample site is considered to be weakly polluted during the period represented by the peatlands profile (Total PAHs with a mean value of 212.3 ng g^−1^). Combined with the analysis of the age-depth model, before 1910, it was unpolluted (mean value: 199.87 ng g^−1^), and after 1980, it was in a weakly polluted degree (mean 243.32 ng g^−1^), which was 34.4 ng g^−1^ higher than that between 1910 and 1980 (mean 208.94 ng g^−1^). This may be due to climate change and human activities affecting the depositional characteristics of PAHs.

## Discussion

### Source identification of BC by δ^13^C_BC_

In this study, the δ^13^C_BC_ value at the surface layer was more negative, while the bottom layer was less negative (varied from − 31.37 to − 26.27‰). Previous studies identified sources of BC in soil included the oxidation of potassium dichromate (K_2_Cr_2_O_7_), thermal oxidation at 375 °C, TOT/RT, BC/TOC ratio analyses, and stable carbon isotope analysis (δ^13^C_BC_) (Gao et al. 2014; Li et al. 2019; Neupane et al. 2020). Among different chemical compositions, δ^13^C_BC_ outperforms other methods and has been widely used (Chen et al. 2013, Qi and Wang 2019). In Altay Mountain peatlands, the BC mainly came from the combustion of C3 plants, while the contribution of C4 plants was relatively small. The morphologies of BC separated from different samples are shown in Figure S1. BC from coal-burning particles (CCPs) (2 cm layer) was usually porous but not always spherical (Figure S1b), which is consistent with previous research results (Accardidey 2003; Wang et al. 2015). In addition, the structure of the 29 cm layer sample was spherical and long-chain, indicating BC was from fossil fuel combustion, in particular automobile exhaust (Figure S1c), in accord with results from other reports (StoffynEgli et al. 1997). These observations supported the idea that the shape of spherical BC was influenced by temperature and degree of combustion as well as the nature of the source material (Rose 1995; Engels et al. 2018). Therefore, the rest samples all represent a hodgepodge of emissions from natural wildfires and BCs that are lumpy or irregular in shape that retained the structure of the plant fibers (porous or tubular, Figures S1a, S1d). As shown in Figures S1a–d, BC in biomass combustion particle (BBP) images were block-shaped, its irregular fragments to (resources) are reported in Zhan et al. (2016) and Min et al (2019), and the primary elements were C and O. SEM analysis not only revealed the morphological characteristics of the three components, but also combined with stable carbon isotope analysis, thus better explained the source of BC. The land vegetation during the current Holocene period in the Altay Mountains is mainly composed of C3 plants, though fossil evidence suggests that C_4_ plants appeared during the Miocene period (Wang et al. 2018). Furthermore, the area has been sparsely populated due to it being primarily forest (especially Taiga Forest Regions), and there were historically no farming areas (Liu 2019). Combined with the age-depth model, the values of δ^13^C_BC_ were lowest during the 1970s, which suggests that biomass combustion was the primary source of BC during this period. Similar results were found from our analysis of PAHs (Table 1).

**Table 1:** δ^13^C_BC_ values from samples from different emission sources

Emission source	Classified	δ^13^C_BC_ (‰)	Reference
C4 grass biomass	Biomass burning	− 15 to − 19‰	Cerling et al. (1997), Saiz et al. (2015)
C3 woody biomass	Biomass burning	− 29 to − 33‰	Cerling et al. (1997)
C3 biomass burning	Biomass burning	− 27 to − 33‰	Bird and Ascough (2012)
Civil coal	Biomass burning	− 23.46‰ ± 0.37‰	Cai (2011)
Coal soot industry	Industry	− 24 to − 26‰	Glaser et al. (2005)
Fireplace soot	Biomass burning	− 26.5‰ ± 0.1‰	Kawashima and Haneishi (2012)
Charcoal	Biomass burning	− 27.4‰ ± 1.7‰	Kawashima and Haneishi (2012)
Diesel vehicle soot	Transportation	− 23 to − 26‰	Lopez-Veneroni (2009)
Liquid fossil fuel (fuel oil)	Transportation	− 27 to − 30‰	Widory (2006)

### Source identification of PAHs by molecular diagnostic ratios

**Fig. 4:**
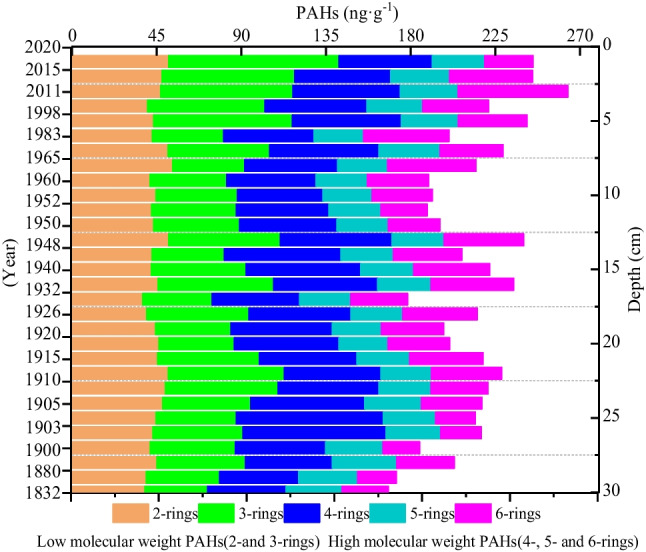
Concentration of each compound of the total polycyclic aromatic hydrocarbons (PAHs) in the peatlands profile

**Fig. 5:**
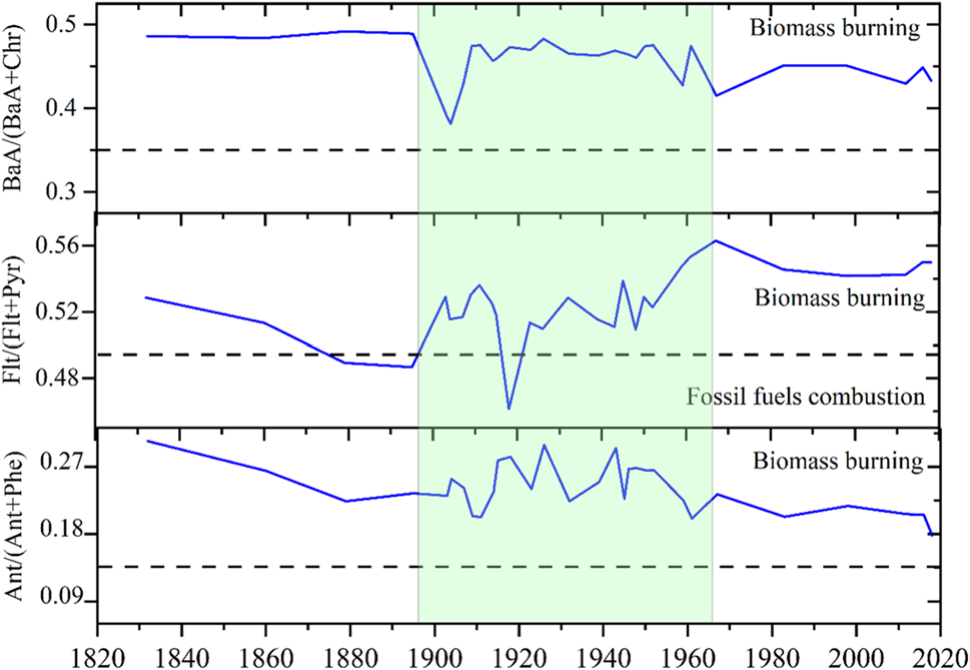
Variation in ratios of BaA/(BaA + Chr), Flt/(Flt + Pyr), and Ant/(Ant + Phe) in Heihu peatland

The diagnostic ratios of PAHs in sediment can be applied to identify the source of combustion products and evaluate the influence of anthropogenic activities on ecosystems (Xing et al. 2011). The data from the Heihu peatland are shown in Figs. 4 and 5 and in Table 2. Figure 4 indicates that each component of PAHs is mainly composed of low ring components with large surface value, and high ring shows that the surface and deep layer are in fluctuations. In permafrost peatlands, the temperature decreased significantly with depth. Therefore, PAHs may accumulate more in the upper active layer than in the lower active layer due to biochemical transformation of organic matter. This may also explain the higher values of PAHs in the surface layer than in the deeper layer. The concentration of PAHs in the soil was used to evaluate pollution level by classifying it into one of the following categories: unpolluted, weakly polluted, moderately polluted, and severely polluted. The sample site has a mean PAH content of 212.3 ng g^−1^ and so can be classified as weakly polluted. Combined with the analysis of the age-depth model, before 1910, it was unpolluted (mean 199.87 ng g^−1^), and after 1980 it was weakly polluted (mean 243.32 ng g^−1^), which was 34.4 ng g^−1^ higher than that between 1910 and 1980 (mean 208.94 ng g^−1^). Based on these data, frequent human activity has intensified the pollution level in the Heihu peatland in recent years.

**Table 2:** Source discrimination index of PAHs

Discriminant index	Value	Average PAHs value in Heihu	Classified
BaA/(BaA + Chr)	< 0.2 0.2–0.35 > 0.35	0.45	Petroleum source Fossil fuel combustion Biomass burning
Flt/(Flt + Pyr)	< 0.4 0.4–0.5 > 0.5	0.52	Petroleum source Fossil fuels combustion Biomass burning
Ant/(Ant + Phe)	> 0.1 < 0.1	0.24	Biomass burning Oil burning
Pyr/BaP LMW/HMW-PAHs ratio	< 1 1–6 < 1 > 1	0.76 1.24	Biomass burning Coal emissions High temperature pyrolysis Fossil fuel combustion

The discriminant source index was obtained from references Yunker et al. (2002) and Gao et al. (2018)

The diagnostic ratios of PAHs help indicate PAH sources. A ratio of Flt/(Flt + Pyr) > 0.5 suggests that PAHs originate from biomass combustion; ratios from 0.4 to 0.5 indicate a combination of biomass and fossil fuel (e.g., diesel, gasoline, and coal) consumption that results in the mixed sources of PAHs. If PAHs were emitted mostly from petroleum sources, both ratios of BaA/(BaA + Chr) and BaA/Chr would be below 0.2; ratios of BaA/(BaA + Chr) > 0.35, Ant/(Ant + Phe) < 0.1 mean the major PAHs sources come from biomass combustion (Yunker et al. 2002; Gao et al. 2018). In this study, the historical variation of PAHs diagnostic ratios in Heihu peatland is shown in Fig. 5. Before the 1900s, BaA/(BaA + Chr) ratios exceeded 0.35 and indicated the primary source of PAH was biomass combustion. From 1900 to 1910, BaA/(BaA + Chr) ratios fell below 0.35 and suggested the primary PAH source was fossil fuel combustion. After the 1910s, the ratios once again surpassed 0.35 (biomass combustion). The Flt/(Flt + Pyr) ratios fluctuated between 0.4 and 0.56, staying below 0.5 before the 1920s but exceeding 0.5 afterward. The Ant/(Ant + Phe) ratios exceeded 0.1 from 1832 to 2019, indicating that the source of pollutants came primarily from biomass combustion. Based on these data, the main sources contribute to pollutant formation in the Heihu peatland of the Altay Mountains: biomass combustion plays the predominant role, followed by fossil combustion.

### Human activities in the Altay Mountains

The increase in BC and PAH concentrations from 1950 to 1980 reflects the development of agriculture and animal husbandry in the Heihu peatland region of the Altay Mountains. Such developments may be attributed to the foundation of “New China” (1949s), during which roads and railways were constructed, and the nation’s transportation industry improved. The tourism industry then developed rapidly, which promoted local economic growth. Consequently, vehicle emissions increased, and the larger population required coal fires for heat and biomass combustion from daily consumption, which resulted in an increase in both BC and PAHs. These activities gradually increased the amount of HMW—PAHs in sediments, though the concentration of BC was reduced after the 1980s (Fig. 3, Figure S2). In addition, despite the increase in industrial coal consumption, construction industry coal consumption, and transportation coal consumption from 2000 to 2010, BC decreased due to the impact of environmental protection policies (Xu 2009; Tang et al. 2016).

Anthropogenic activities are the main source of PAHs, though PAH pollution may also have natural origins such as volcanic eruptions and forest fires (Ma et al. 2021). As previously mentioned, a PAH concentration of 0 to 10 ng·g^−1^ in the soil reflects natural pollution (Edward 1983). However, the range of PAHs in the peatland core samples we analyzed was between 168.09 and 263.53 ng·g^−1^ (Fig. 3), indicating influences from human activities. After 1980, Xinjiang’s economy developed rapidly and the use of fuel (especially the use of petrochemical fuel) entered a period of rapid growth. Furthermore, the GDP, population, and the number of tourists in Altay increased rapidly after 1990. During this period, PAHs in sediments increased rapidly, and HMW—PAHs also increased significantly. The Heihu peatlands are located in a tourist attraction area, and from the early 1980s to the mid-1990s, the Kanas tourist area transformed from spontaneous tourism to government-led tourism (China 2009; Region 2012). Local transportation infrastructure was also improved, particularly with regard to the construction of the Beitun-Fuyun highway and the Buerjin-to-Kanas airport (Ma 2018). Approximately 3 billion Yuan has been invested in capital construction in the region, and local infrastructure and supporting facilities have additionally been increasingly improved. At a similar site, Shen et al., (2016) found that PAHs in a sediment core column collected from Bosten Lake primarily consisted of LMW-PAHs. HMW-PAHs began to appear slowly and fluctuated from 1950 to 1980, though they have rapidly increased since the end of 1980, which is consistent with the results of this paper.

### Correlations among physicochemical parameters, BC, and PAHs

The physicochemical properties of soil significantly influence the soil BC content and PAH accumulation (Hu et al. 2017; Min et al. 2019). Combined with the Spearman correlation analyses (Figure S3), Briefly, there were significantly negative correlations between TOC and 5-ring. the BC in Heihu peatlands core was significantly positively correlated with BC/TOC, TOC (correlation coefficients were 0.91 and 0.546, respectively), the correlation reached significance at 0.01 level. This suggests that BC and TOC in the same layer may have the same source. BC and the TOC bound to it remain in the environment for hundreds of years because of the chemically and biologically inert nature of BC (Xu et al. 2017). Moreover, BC has a high sorption capacity because of its large surface area to volume ratio. Therefore, it adsorbs and immobilizes TOC and clay minerals and stabilizes and retains them (Zhan et al. 2016). TOC was negatively correlated with total PAHs and was negatively correlated with 5-rings. HD was positively correlated with BC but negatively correlated with PAHs (Table S3). This can be explained by the reasons are the larger HD humus represents high organic matter content, which can not only increase the adsorption of PAHs but also reduce the mobility and degradation of PAHs (Ping and Luo 2005; Yu et al. 2023).

There is significant positive correlation among the 2-, 3-, 4-, 5-, 6-rings, LMW, HMW, and total PAHs, which suggest that these compounds originated from the same source. There was no significant correlation between PAHs and BC in general. The difference in correlation was mainly caused by the similarities and differences in sources of PAHs and BC. Both PAHs and BC came from biomass combustion before 1910, the correlation between PAHs and BC in this period was strongly positive correlation. From 1910 to 1980, both PAHs and BC come from the burning of fossil fuels, they have weak positive correlation. However, after 1980s, PAHs mainly came from coal combustion while BC came from biomass combustion, and the two did not have homology, so they have a negative correlation. In general, high-ring PAHs are mainly derived from various combustion processes (especially fossil fuel combustion), so the significant correlation between BC and 5-ring PAHs is evidence that both BC and PAHs were derived from fossil fuel combustion after 1980.

### Comparison of worldwide

BC is an important link in the global carbon cycle (Hammes et al. 2007). BC content in the atmosphere reached − 2 µg C·m^−3^ in Beijing and Shanghai, nearly 2.5 times higher than that in Gosan (Korea) (Chen et al. 2013). Compared with BC content in other areas, the BC in Heihu peatlands core was similar to those in other areas (Daxinganling, Sanjiang Plain, Zoige Plateau; Table S4). Due to different geographical locations and altitudes, variations of BC in the peatlands obviously differ. The Zoige region is located on the northeast edge of the Qinghai Tibet Plateau, the largest plateau wetland in the world. Because it intersects the China monsoon region and the Qinghai Tibet plateau region, this area is very sensitive to climate change. The total organic carbon range for the Huahu peatlands core was 54.44–142.78 mg g^−1^. BC in the peatlands core far exceeds that in coastal environment sediments. Peatland sediment is rich in total organic carbon, and other physical and chemical properties of peatland soil may be at play (Su et al. 2017). The BC content in the Great Khingan area is low due to the altitude. In high-altitude areas, fires enhance BC, resulting in the distribution of BC reserves in the Great Khingan area that were only affected by natural fires, a finding more pronounced in the middle but less so in the north and south (He et al. 2016). Therefore, BC is not just affected by human activities. Climate factors such as topography, latitude, altitude, and temperature also affect BC amounts.

Compared with other studies of peatlands in China and around the world, the concentrations of PAHs in this study were considerably lower (Table S5), most likely because of the remote and isolated location of this region. the total amount of PAHs in Heihu peatlands (212.25 ng·g^−1^) is lower than that in Swiss peatlands, and the total PAHs in Swiss peatlands (387.3–2853 ng·g^−1^) exceeds the American EPA standard and is much higher than that in other typical wetland sediments. Furthermore, compared with the content of PAHs in other swamp sediments in Sanjiang Plain Gao et al. (2018), the content of PAHs in this study is also relatively low, which is equivalent to the total PAHs of Motianling peatlands in Daxinganling. The results differed slightly from the results reported by Ming et al. (2010), which they found that the PAHs value in a peatland core profile from Zoige Hongyuan County was 36.2–408 ng·g^−1^. The main reason is that peatland is very sensitive to global climate change, and the increase of regional temperature may affect the deposition and accumulation of PAHs in peatlands. Our results also conflicted with Cui et al. (2015) who reported that PAHs varied from 207 to 611 ng·g^−1^. From 1952 to 2012, industrialization and coal development significantly influenced PAHs, which gradually increased and maximized in 2012 (263.53 ng·g^−1^). PAHs decreased after 2012 due to the use of clean energy. Through the above analysis results, it can be seen that Heihu peatlands have been disturbed by human beings, and the environmental pollution is more obvious.

## Conclusions

A sediment core sample collected from the Heihu peatland established a profile that dates to the 1830s. Analyses of intervals of core sample reveal that during the period from 1832 to 2018, BC and δ^13^C_BC_ values decreased, while PAHs increased. BC increased before 1910 with an average of 59.85 mg g^−1^. The total PAHs increased before 1910, but generally decreased from 1910 to 1980, with a low concentration of 178.35 ng·g^−1^ which indicates that the peatlands were weakly polluted. The δ^13^C_BC_ value shows that BC comes from biomass combustion, especially C_3_ plant combustion. Moreover, the ratios of BaA/(BaA + Chr), Flu/(Flu + Pyr) suggest that PAHs have mainly originated from the biomass and the burning of fossil fuel. From 1832 to 2018, the changes in BC and PAH concentrations reflect three different periods of traditional agriculture and animal husbandry, industrial development, and emission reduction policy formulation in Altay. This study will help the Chinese government to achieve sustainable development in similar ecological regions.

### Supplementary Information

Below is the link to the electronic supplementary material.

Supplementary file1 (DOCX 1037 KB)
