# Supplementary material for: Investigation of 200 anthropogenic activities in a representative alpine peatland in the Altay Mountains, northwestern China
Source: Environ Sci Pollut Res Int. 2024 May 6;31(23):34558–68. doi: 10.1007/s11356-024-33498-1 (PMC11136768; doi:10.1007/s11356-024-33498-1)
Supplement: Supplementary file 1 — Supplementary file1 (DOCX 1037 KB) [file 11356_2024_33498_MOESM1_ESM.docx]

**Support information**

**Investigation of 200-** **anthropogenic activities in a representative peatland in** **the Xinjiang Altay Mountains, northwestern China**

Nana Luo*^a^*, Rui Yu*^a^*, Bolong Wen*^a^*^*^, Xiaoyu Li*^a^*, Qilin Zhang*^a^*^,^ *^b^*, Xiujun Li*^a^*

*a* Key Laboratory of Wetland Ecology and Environment, Northeast Institute of Geography and Agroecology, Chinese Academy of Sciences, Changchun 130102, China

*b* University of Chinese Academy of Sciences, Beijing, 100049, China

*Corresponding author: [wenbolong@iga.ac.cn](mailto:wenbolong@iga.ac.cn)

The authors declare no competing financial interest.

Number of pages: 10

Number of tables: 5

Number of figures:3

## Table S1. Average concentrations (ng·g^-1^) and standard deviations of PAHs in different periods.

|  | Before 1910 (n=4) | | 1910-1980(n=21) | | After 1980 (n=5) | |
| --- | --- | --- | --- | --- | --- | --- |
| Composi-tions | Average (ng·g^-1^) | Standard Deviation | Average (ng·g^-1^) | Standard Deviation | Average  (ng·g^-1^) | Standard Deviation |
| NAP | 12.06 | 1.57 | 14.03 | 3.19 | 11.51 | 1.04 |
| ANY | 7.93 | 0.07 | 8.02 | 0.48 | 8.14 | 0.13 |
| ANA | 9.07 | 0.89 | 9.66 | 1.24 | 10.43 | 0.84 |
| FLU | 12.16 | 1.88 | 13.50 | 2.05 | 15.83 | 1.89 |
| PHE | 25.00 | 6.08 | 29.41 | 6.77 | 49.03 | 7.15 |
| ANT | 8.42 | 0.68 | 9.46 | 1.33 | 12.30 | 0.73 |
| FLT | 7.63 | 0.69 | 8.68 | 0.81 | 12.17 | 1.56 |
| PYR | 7.51 | 0.48 | 7.88 | 0.63 | 10.11 | 1.20 |
| BaA | 7.89 | 0.45 | 7.98 | 0.44 | 8.46 | 0.30 |
| IcP | 23.84 | 4.84 | 34.47 | 6.16 | 39.98 | 10.96 |
| CHR | 8.29 | 4.53 | 10.15 | 0.66 | 10.68 | 0.74 |
| BbF | 11.37 | 0.73 | 11.33 | 1.45 | 11.50 | 0.64 |
| BKF | 9.49 | 9.23 | 18.34 | 4.43 | 13.20 | 4.17 |
| BeP | 11.60 | 0.61 | 10.88 | 0.62 | 11.74 | 0.76 |
| BaP | 12.00 | 1.12 | 10.36 | 0.71 | 11.78 | 0.98 |
| DhA | 7.78 | 0.62 | 6.45 | 0.37 | 6.46 | 0.14 |

Abbreviations of PAHs (number of rings are reported in brackets): NaP, Naphthalene (2); Any, Acenaphthylene (3); Ana, Acenaphthene (3); Flu, Fluorene (3); Phe, Phenanthrene (3); Ant, Anthracene (3); Flt, Fluoranthene (4); Pyr, Pyrene (4); Chr, Chrysene (4); BaA, Benzo(a) anthracene (4); BbF, Benzo(b)fluoranthene (5); BkF, Benzo(k)fluoranthene (5); BeP, Benzo(e)pyrene (5); BaP, Benzo(a)pyrene (5); DhA, Dibenz[a,h] anthracene (5); IcP, Ideno(1,2,3-cd) pyrene (6).

## Table S2. Definition of PAHs pollution degree

| PAHs（ng·g^-1^, Dry) | Pollution level |
| --- | --- |
| <200 | Unpolluted |
| 200-600 | Weakly polluted |
| 600-1000 | Moderately polluted |
| >1000 | Severely polluted |

## Figure S1. SEM images of BC examples from different samples. All scale bars represent 50 μm. a) panel represents BC from biomass burning particles (BBP), b) represents coal combustion particles (CCP), c) represents automobile exhaust (AE), d) represents wood burning, e) shows the major elemental composition, h) shows EDX results of BC from TSP samples.


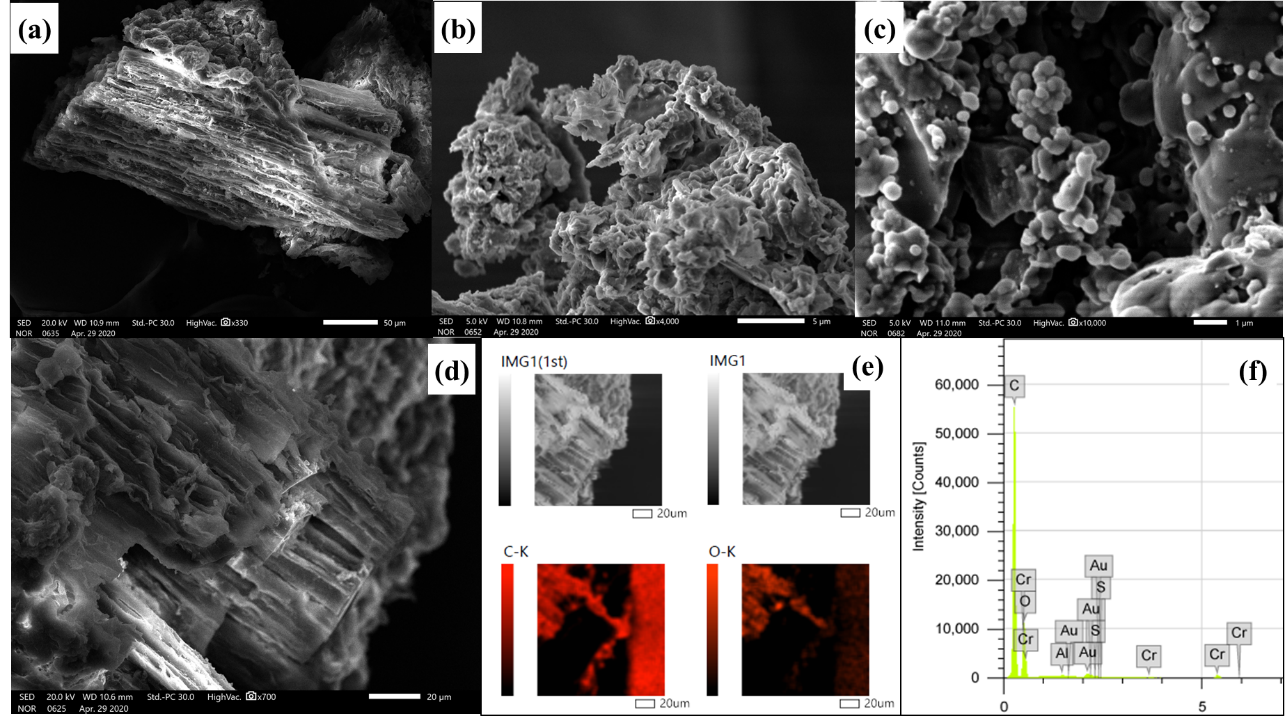


## Figure S2. a) Construction Industry coal consumption (red line); b) Construction Industry coal consumption (Origin line); c) Transportation (blue line), d) Variation of BC (black line) from 1832 to 2018 in Heihu peatlands.

## Figure S3. Correlation between BC and important index in Heihu peatlands.

## Table S3. Spearman’s correlation coefficients for BC and PAHs in Heihu peatlands.

| Time age | correlation coefficients |
| --- | --- |
| Before 1910 | 0.952** |
| 1910-1980 | 0.268 |
| After 1980 | -0.5 |

** With a significance level of 0.01

## Table S4. The BC Contents (mg g-1) for different deposition archives.

| Types | Description | Contents (mg g^-1^) |
| --- | --- | --- |
| Peatland | Altai Mountain, China | 11.7-67.5 |
| Peatland | Sanjiang Plain, China | 3.2-61.2 |
| Peatland | Great Khingan, China | 6.22-75.61 |
| Peatland | Zoige Plateau, China | 40.1-82.27 |
| Forest soil | Amazon, Brazilian | 1-27 |
| Loess | Loess Plateau, China | 1-5 |
| Lake sediments | Taihu, China | 0.43-1.95 |
| Lake sediments | Tibetan Plateau, China | 0.49-1.09 |
| Cintinental shell (near urbon) | Palos Verdes Shelf, American | 1.2 |

## Table S5. The PHAs concertation (ng g^−1^) of peatland for previous works and this study.

|  | Location | PHAs (ng g^-1^) | Reference |
| --- | --- | --- | --- |
| 1 | Altay mountain (Heihu) | 168.09 - 263.53 | This study |
| 2 | Athabasca, Canada | 700-12000 | (He et al., 2019) |
| 3 | Jura Mountains, Switzerland | 387.3-2853.1 | (Berset et al., 2001) |
| 4 | Peatlands of Eastern Canada | 48 - 2790 | ([Dreyer et al. 2005](#_ENREF_1)) |
| 5 | Peatlands of northeast Poland | 70 - 439 | ([Malawska et al. 2002](#_ENREF_3)) |
| 6 | Northeastern of China  (Motianling of Great Hinggan Mountains) | 1874.4-5040 | (Shi et al., 2007) |
| 7 | Northeastern of China  (Changbai Mountains) | 1404-1936 | (Shi et al., 2008) |
| 8 | Northeastern Qinghai Tibetan Plateau, Zoige Peatland | 27.5-726.9 (Mean: 318.98) | ([Yang et al. 2021](#_ENREF_4)) |
| 9 | Sanjiang Plain swamp | 3654 - 975.4 | ([Zhao et al. 2010](#_ENREF_5)) |
| 10 | Zhalong wetland | 31.9 - 290 | ([Liu et al. 2011](#_ENREF_2)) |

# References

Dreyer, A., C. Blodau, J. Turunen, and M. Radke. 2005. The spatial distribution of PAH depositions to peatlands of Eastern Canada. Atmospheric Environment **39**:3725-3733.

Liu, J. W., Q. L. Xie, and Y. Wang. 2011. Pollution characteristics of polycyclic aromatic hydrocarbons in surface sediments of Zhalong Wetland. Environmental l Science **32**:5.

Malawska, M. G., I. Bojakowska, and B. A. Wi Komirski. 2002. Polycyclic aromatic hydrocarbons (PAHs) in peat and plants from selected peat‐bogs in the north‐east of Poland. Journal of Plant Nutrition & Soil Science **165**:686–691.

Yang, Z. a., Q. Su, H. Chen, and G. Yang. 2021. Anthropogenic impacts recorded by a 200-year peat profile from the Zoige Peatland, northeastern Qinghai-Tibetan Plateau. Catena **206**.

Zhao, H., M., J. Wang, X. F. Yu, X. Lv, G., and G. P. Wang. 2010. Distribution of polycyclic aromatic hydrocarbons (PAHs) in swampy soil and effects of fire in Sanjiang Plain. Wetland Science.
